# Temporal Dynamics and Heterogeneity in Brain Metastases: A Single-Center Retrospective Analysis of Vulnerabilities in Current MRI Surveillance Practices

**DOI:** 10.3390/medicina62010187

**Published:** 2026-01-16

**Authors:** Claudia Tocilă-Mătășel, Sorin Marian Dudea, Gheorghe Iana

**Affiliations:** 1 Department of Radiology, Faculty of Medicine, Iuliu Hatieganu University of Medicine and Pharmacy, 400012 Cluj-Napoca, Romania; 2 Medima Health SA, 060254 Bucharest, Romania

**Keywords:** brain metastases, longitudinal tracking, lesion evolution, imaging variability, radiology reporting, neuro-oncology

## Abstract

*Background and Objectives*: Brain metastases frequently evolve over time in multiple waves, especially in patients with prolonged survival. Despite repeated imaging and targeted therapies, lesion-level continuity is fragmented in clinical practice, as follow-up is typically limited to pairwise MRI comparisons. The aim of the study is to assess the ability of routine narrative MRI follow-up reports to preserve longitudinal lesion identity and to reconstruct a coherent trajectory of disease evolution. *Materials and Methods*: We conducted a single-center, retrospective, observational study of all brain MRI examinations performed between June 2024 and June 2025 (n = 731 scans, 616 patients). All imaging reviews and longitudinal lesion tracking were performed by one board-certified neuroradiologist. Adult patients with confirmed brain metastases and at least three MRI examinations (including external studies) were included. We assessed the concordance of routine narrative MRI follow-up reports against a longitudinal review of all available MRIs and treatment timelines, which served as the reference standard. Lesion identity was considered preserved when reports explicitly recognized and linked lesions across time points, and lost when identity was omitted or ambiguous in at least one report. *Results*: The final cohort comprised 73 patients (477 tracked lesions). More than half of monitored lesions disappeared (42.9%) or evolved into post-treatment sequelae (9.9%), and were omitted from narrative reports, limiting retrospective recognition without prior imaging. The ability of routine reports to preserve lesion identity declined as cases became more complex. Concordance was higher in uniform evolution patterns (≈60%) but dropped to 18.2% in mixed evolution. A similar decline was seen with sequential metastatic waves, defined as new metastases appearing at distinct time points: 65.2% (1 wave), 46.7% (2 waves), 18.2% (3 waves), and complete loss of continuity when >3 waves occurred. *Conclusions*: Routine narrative MRI follow-up reports generally provide adequate information in simple cases with uniform lesion behavior, but tend to lose critical details as disease trajectories become more complex, particularly in heterogeneous or multi-wave disease. Even when individual lesions are identified across examinations, documentation remains fragmented and reflects only a snapshot of the disease course rather than an integrated longitudinal perspective. These findings highlight a critical vulnerability in current follow-up practices. Improving lesion-level continuity, potentially through AI-assisted tools, may enhance the accuracy, consistency, and clinical utility of MRI surveillance in patients with brain metastases.

## 1. Introduction

Brain metastases are a common and severe complication of systemic cancers, arising from the hematogenous dissemination of malignant cells to the brain parenchyma, most commonly originating from lung cancer, breast cancer, and melanoma. They are frequently associated with poor prognosis [1]. However, advances in oncology have significantly prolonged survival in many patients, leading to an increasing incidence of brain metastases. In modern clinical practice, brain metastases often appear in multiple waves over time. Early detection through MRI and precise treatment, most commonly with stereotactic radiotherapy, have greatly improved patient outcomes [2]. As a result, patients frequently undergo several rounds of targeted treatment for new metastatic lesions that arise at different time points [3].

Given the temporal spacing between metastatic episodes, patients often switch between treatment or imaging centers. Medical records are frequently fragmented, inconsistently structured, or incomplete, particularly in cross-institutional care. In routine clinical neuro-oncology practice, follow-up imaging is often limited to pairwise comparisons of only the most recent two or three MRI scans, despite patients having much longer histories of disease and treatment. The high volume of oncologic imaging, combined with variability in radiology reporting, further compounds the difficulty of achieving a coherent, longitudinal view of disease evolution [4].

In this context, the routine narrative MRI follow-up report plays a central role in clinical decision-making. These reports guide oncologists and neurosurgeons in determining whether lesions are new, progressive, stable, or resolved, and whether additional treatment is needed. The intended clinical use of the narrative MRI follow-up report is therefore to preserve lesion identity and evolution across time points and to provide an accurate and continuous record of disease evolution. However, in practice, the narrative report is fragmented: it typically consists of a pairwise comparison between two consecutive examinations rather than an integrated longitudinal perspective. This segmented approach represents only a snapshot of the disease course, and may lead to underestimation of treatment response or misclassification of progression. These challenges have led to initiatives aimed at standardizing imaging reporting in neuro-oncology, including structured reporting systems (such as RANO-BM), which are designed to improve the consistency of lesion descriptions. However, the applicability of RANO-BM can be challenged in the setting of complex metastatic disease, particularly when lesions evolve asynchronously or exhibit treatment-related changes such as pseudoprogression or radiation effects, which complicate longitudinal tracking [5].

Advances in artificial intelligence have shown promising potential for longitudinal lesion tracking and for integrating multi-temporal imaging data, with the ability to maintain lesion identity across multiple time points [6]. By identifying where longitudinal information is most frequently lost in clinical practice, either because lesions disappear from narrative reports or become difficult to recognize on imaging, our study highlights specific gaps that AI-guided approaches could address to improve follow-up monitoring.

The objective of this study is to assess the ability of routine narrative MRI follow-up reports to preserve longitudinal lesion identity and to reconstruct a coherent trajectory of disease evolution. The study was designed to descriptively quantify longitudinal reporting continuity rather than to test a therapeutic hypothesis. A neuro-radiologist’s longitudinal review of all available imaging served as the reference standard.

## 2. Materials and Methods

**Figure 1:**
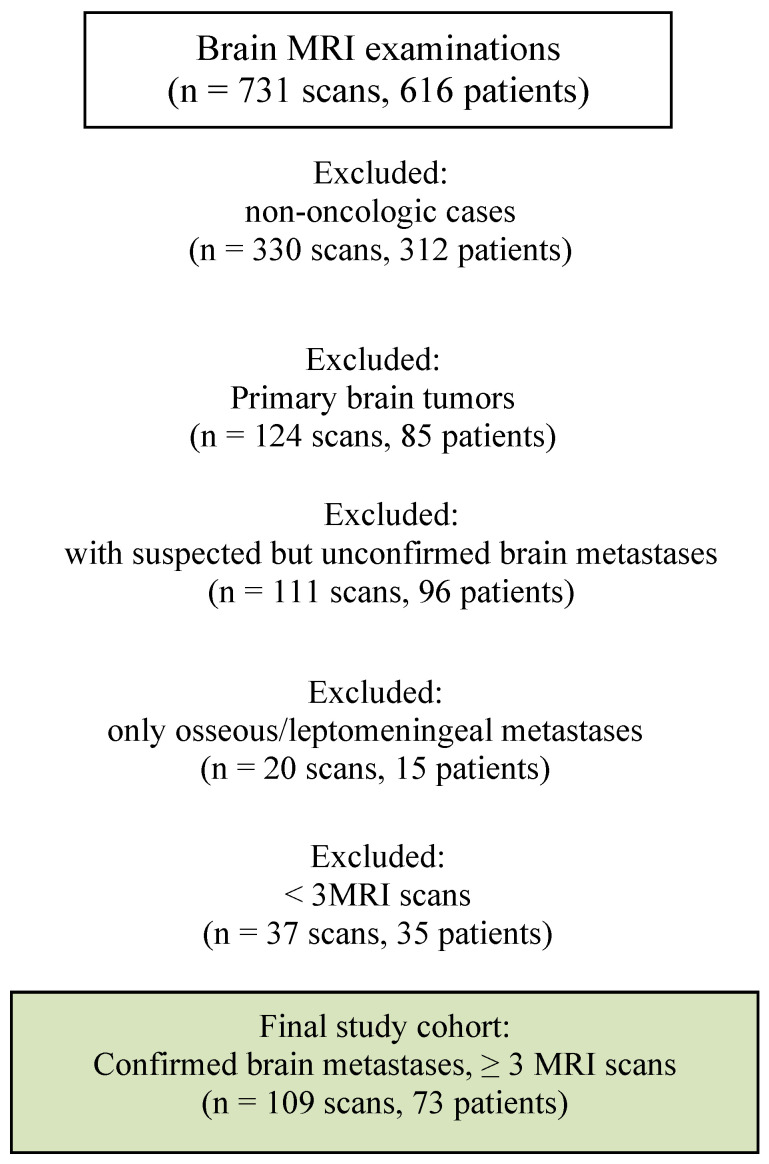
Selection process of brain MRI examinations and exclusions leading to the final study cohort.

We conducted a single-center, retrospective, observational study. The screening set comprised all brain MRI examinations interpreted between June 2024 and June 2025 (n = 731 examinations, 616 patients) by a board-certified radiologist trained in neuroradiology with a focus on neuro-oncology imaging, currently in the second year of independent clinical practice. These examinations were reviewed to identify adult patients with confirmed cerebral metastases, defined as well-demarcated enhancing lesions on post-contrast T1 with or without perilesional edema, in patients with known systemic malignancy. Inclusion criteria were at least one MRI performed at our center during the index year, a histologically proven systemic malignancy, and MRI findings consistent with brain metastases in the appropriate clinical context. Histopathologic confirmation was available only in a minority of surgically resected brain metastases. Exclusion criteria included 330 examinations in 312 patients with non-oncologic neurological complaints such as dizziness, vertigo, suspected stroke or headache, 124 examinations in 85 patients with primary brain tumors, 111 examinations in 96 patients with suspected but unconfirmed brain metastases, 20 examinations in 15 patients with exclusively osseous or leptomeningeal metastases, and 37 examinations in 35 patients with fewer than three available MRI studies, including external ones. After applying these criteria, the final study cohort comprised 109 examinations in 73 adult patients (Figure 1). All included patients were adults.

### 2.1. Longitudinal Data Collection

We used the 12-month interval solely as a screening window for patient identification, after which we retrospectively reconstructed the complete longitudinal imaging history for each included patient. For this purpose, all available brain MRI examinations were retrieved, including studies performed at our center as well as external examinations provided by patients and subsequently imported into institutional PACS viewer as part of routine clinical workflow. In total, 422 MRI examinations were included in the longitudinal analysis. Of these, 109 belonged to the index screening period, while the remaining examinations consisted of earlier MRIs, which were required to assemble each patient’s full longitudinal record. Consequently, many of these examinations were not part of the initial 731-scan screening set and fell outside the 12-month time window.

### 2.2. MRI Acquisition at Our Institution

All MRI examinations performed at our center were acquired on a 3T MRI system (GE Signa Pioneer, GE Healthcare, Milwaukee, WI, USA) equipped with a 21-channel phased-array head and neck coil. The standard post-contrast sequence used for lesion assessment was a 3D T1 FSPGR acquisition with 1 mm isotropic voxels and no interslice gap.

### 2.3. External MRI Examinations

The remaining examinations were performed at multiple external centers using heterogeneous MRI systems and acquisition protocols. Although detailed sequence parameters (e.g., TR/TE or contrast timing) were not uniformly available, all external studies included at least one high-resolution post-contrast T1-weighted sequence with a slice thickness ≤ 1.5 mm.

### 2.4. Lesion Identification and Tracking

All imaging reviews and longitudinal lesion tracking were performed by a single board-certified neuroradiologist. This approach was chosen to maximize internal consistency of lesion identification across multiple time points, acknowledging that it may limit inter-observer generalizability. Lesions were identified on post-contrast T1-weighted images and correlated with all available sequences in the protocol. For each lesion, the following outcomes were documented: new appearance, disappearance, dimensional regression, dimensional stability, dimensional progression, post-treatment sequelae (non-enhancing residual changes), and surgical removal. A dimensional change was considered progression if both the largest axial diameter and the perpendicular diameter increased by ≥1 mm, regression if both decreased by ≥1 mm, and stable if one or both axes changed by <1 mm. Measurements were performed manually on post-contrast T1-weighted images by the reviewing radiologist. Dimensional changes were defined conservatively to detect directionality rather than absolute magnitude, consistent with prior approaches in metastatic lesion monitoring. Lesion identity was maintained across all available time points to ensure continuity of tracking.

### 2.5. Assessment of Reporting Consistency

All available radiology reports (including external reports when available) were reviewed for longitudinal consistency. Of the 422 examinations, reports were available for 304 (72%); 28% were either missing or only partially accessible (e.g., limited to the conclusion section). Missing or incomplete reports reflected the real-world availability of clinical documentation rather than study-related loss of data. These examinations were not excluded; instead, reporting gaps were incorporated into the continuity assessment, as they represent an integral part of the phenomenon under investigation. For each included patient, reports were available for more than half of the performed examinations, ensuring adequate longitudinal assessment. For the purpose of this study, an incomplete or ambiguous report was defined as a follow-up MRI report in which lesion-level longitudinal tracking was not possible due to one of the following scenarios:

- Omission: a previously treated lesion (often visible only as post-treatment sequelae and no longer enhancing) was absent from the report, making it impossible to recognize its prior metastatic nature without access to historical imaging. Other causes for omission included lesions no longer detectable on current scans or identifiable only by reviewing prior imaging.
- Ambiguous lesion identity: inability to determine whether a lesion corresponded to a previously reported metastasis, due to changes in size, post-treatment morphological alterations (including the merging of two previously separate treated lesions), or insufficiently precise location descriptions. Another situation was nonspecific statements without lesion-level detail (e.g., “overall regression” or “disease progression”), sometimes without clarifying which lesions were old and treated but still enhancing versus new.

Reporting concordance was assessed at the patient level to reflect the clinical reality in which follow-up decisions are made based on the overall interpretation of a patient’s disease status rather than individual lesions. Reports were considered concordant if lesion identity was preserved across all available examinations for that patient (explicit recognition and linkage to prior states), and discordant if lesion identity was lost in at least one report, either through omission or ambiguity. Partial preservation of lesion identity was not classified separately; if lesion identity was lost for at least one lesion at any time point, the patient was categorized as discordant. The reference standard was a longitudinal review of all MRI examinations and treatment timelines, performed by a board-certified neuroradiologist.

This retrospective study was conducted in compliance with Good Clinical Practice (GCP) guidelines, including the principles of ICH E6(R3), with approval and oversight from the Institutional Ethics Committee, ensuring patient confidentiality and data integrity.

### 2.6. Analysis

The analysis quantified reporting concordance, defined as the ability of routine narrative MRI reports to preserve longitudinal lesion identity and to reconstruct a coherent trajectory of disease evolution compared with the longitudinal review of all available MRIs and treatment timelines, which served as the reference standard. Concordance rates with 95% confidence intervals were calculated using Wilson’s method with patient-level cluster-robust variance. Patient-level concordance was selected as the primary analytic unit because clinical decisions are guided by the overall assessment of disease evolution rather than isolated lesion-level descriptions. Lesion-level tracking was used to inform this assessment, but patient-level concordance better reflects real-world reporting impact. All analyses were performed using Python (version 3.11 64-bit) [7], using standard statistical libraries.

## 3. Results

### 3.1. Temporal Evolution of Brain Lesions

Across the 73 included patients, a total of 422 MRI examinations were available for longitudinal tracking, corresponding to a median of 4 MRI scans per patient (range 3–16; mean 5.8).

A total of 477 brain metastases were longitudinally tracked in these patients, each of whom had at least three MRI examinations (including external studies).

**Figure 2:**
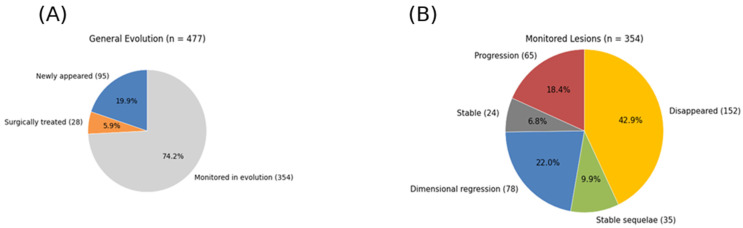
(**A**) Distribution of all 477 tracked brain metastases, including newly detected lesions at last follow-up (n = 95; 19.9%), surgically treated lesions (n = 28; 5.9%), and monitored lesions (n = 354; 74.2%). (**B**) Detailed classification of the 354 monitored lesions into disappeared (n = 152; 42.9%), stable sequelae (n = 35; 9.9%), regression (n = 78; 22.0%), stable (n = 24; 6.8%), and progression (n = 65; 18.4%).

Of the 477 lesions, 95 (19.9%) were newly detected at the last follow-up scan, 28 (5.9%) were surgically treated, and 354 (74.2%) were monitored over time (Figure 2A). Among the monitored lesions, evolution was heterogeneous: 152 (42.9%) disappeared from imaging; 35 (9.9%) evolved into sequelae; 78 (22.0%) regressed; 24 (6.8%) remained stable; and 65 (18.4%) demonstrated dimensional progression (Figure 2B).

Disappearing lesions were typically previously treated and non-enhancing, and without access to prior imaging could not be confidently localized or recognized as treated disease. This limitation becomes particularly relevant when post-treatment changes, such as radiation necrosis, occur later at the same site. In this classification, sequelae referred to non-enhancing post-treatment residues, regression indicated dimensional decrease while maintaining residual contrast enhancement, stability reflected unchanged size and enhancement, and progression represented measurable dimensional increase.

**Figure 3:**
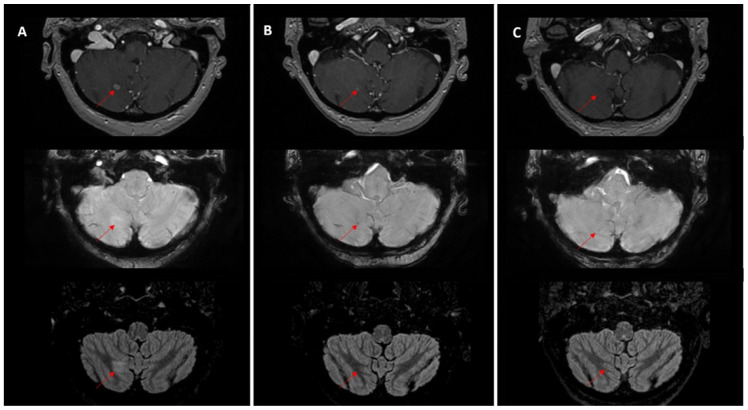
Evolution of a right cerebellar metastasis (red arrows) in a patient with breast cancer and multiple secondary lesions. (**A**) Before treatment MRI showed a 6 mm right cerebellar lesion with ring enhancement on post-contrast T1, no susceptibility artifacts on SWAN and hyperintense on T2/FLAIR. (**B**) At 8 weeks after whole-brain radiotherapy (30 Gy/10 fractions) with a sequential boost up to 45 Gy to the cerebellar lesion, there was dimensional regression with residual millimetric enhancement, the appearance of a hypointense susceptibility artifact on SWAN, and resolution of signal abnormalities on FLAIR. (**C**) At 6 months after radiotherapy, the lesion was no longer visible on conventional sequences; only a subtle punctiform hypointense focus on SWAN could be appreciated, which would not be recognized as a treated metastasis in the absence of longitudinal tracking.

More than half of the monitored lesions (52.8%; 187/354) either disappeared from imaging or evolved into stable sequelae (Figure 3), representing prior sites of treated disease. In the absence of access to historical imaging, these lesions could not be reliably localized or recognized as metastases, particularly when later affected by post-treatment changes such as radiation necrosis. This highlights a key vulnerability in routine follow-up: the potential loss of lesion identity over time, which can contribute to underreporting or misclassification in narrative radiology reports.

**Figure 4:**
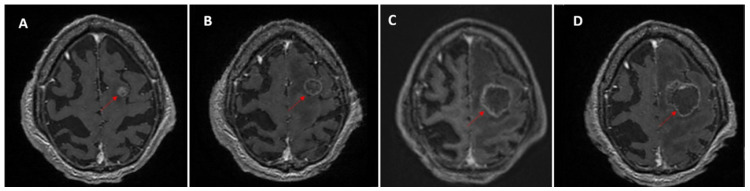
Longitudinal MRI of a left frontal brain metastasis (red arrows) in a patient with lung cancer treated with stereotactic radiotherapy (SRT, 35 Gy/5 fractions). (**A**) Baseline scan before SRT shows an enhancing lesion. (**B**–**D**) Post-treatment follow-up demonstrates dimensional increase with irregular, thin margins and persistent enhancement over time.

A subset of monitored lesions, 18.4% (65/354), showed dimensional increase during follow-up. Without histopathologic confirmation or advanced imaging criteria, it is difficult to differentiate true tumor progression from post-treatment effects such as pseudoprogression or radiation necrosis. When reports describe only dimensional increase without noting that the lesion was irradiated (e.g., three or six months earlier), important clinical context is lost, potentially creating interpretative ambiguities. In such cases, post-treatment enlargement may be misclassified as progressive metastatic disease rather than a treatment-related change (Figure 4).

**Figure 5:**
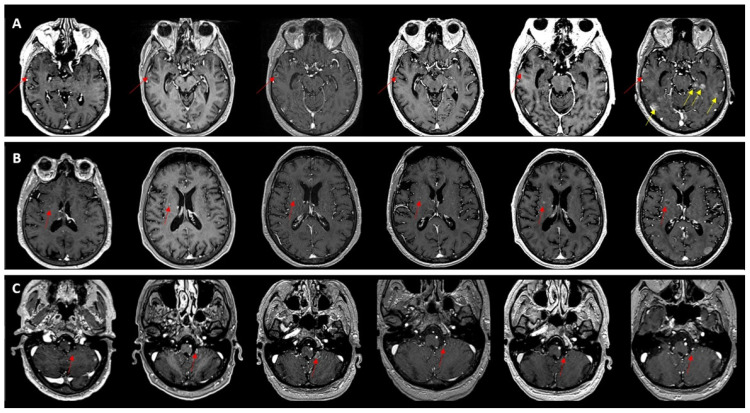
Longitudinal MRI follow-up of a patient with lung cancer and complex treatment history, showing brain metastases evolving in multiple “waves.” Each row illustrates a different lesion (**A**–**C**). (Row (**A**)) A right temporal metastasis (red arrows) shows slight growth over the first three examinations, loss of enhancement at the fourth, followed by renewed enlargement and contrast uptake, along with the appearance of additional new metastases (yellow arrows). (Row (**B**)) A right lentiform nucleus lesion (red arrows) becomes more conspicuous on the third examination, subsequently regresses, and then shows renewed growth, remaining ambiguous between treatment effect (necrosis) and true progression. (Row (**C**)) A left cerebellar lesion (red arrows) visible on the third examination regresses after treatment. This case highlights the heterogeneity of longitudinal evolution, with coexisting progression, regression, and emergence of new lesions.

Lesions that remained stable (6.8%; 24/354) or showed regression (22.0%; 78/354) were typically previously treated but retained some degree of contrast enhancement. In clinical follow-up, these lesions may be more difficult to interpret when new metastases appear, particularly if their treated status or precise anatomical location is not explicitly documented in the report (Figure 5). Without consistent lesion-level tracking and explicit mention of prior treatment, residual post-treatment changes could be mistaken for newly developed or recurrent disease, contributing to potential reporting inconsistencies.

### 3.2. Dimensional Evolution Patterns and Reporting Concordance

Based on dimensional changes observed across the entire cohort (n = 73 patients) during follow-up, patients were classified into four lesion evolution categories: uniform regression—all lesions showed dimensional decrease or disappeared; uniform progression—all lesions showed dimensional increase; new lesion wave—appearance of additional brain metastases during follow-up, regardless of the behavior of pre-existing lesions; mixed evolution—coexistence of lesions with different dimensional trends (regression, stability, and/or progression) (Table 1).

In the uniform regression group, incomplete reports most frequently contained generic statements such as “overall regression” or “no residual enhancement”. Treated lesions that had disappeared or evolved into sequelae were often omitted entirely, making longitudinal tracking dependent on access to prior imaging.

In the uniform progression group, ambiguities commonly involved merging of previously treated lesions, likely due to post-radiotherapy necrosis, or uncertainty about whether they appeared simultaneously or sequentially, and whether both had been treated. In some cases, residual contrast enhancement of treated lesions was accompanied by new lesion appearance treated in a second wave, but without clear specification of treatment history or chronological appearance. Lesion localization was sometimes vague (e.g., “in the frontal lobe”) and treatment references non-specific (e.g., “treated brain lesions”).

In the new lesion wave group, the narrative frequently shifted focus to the newly detected enhancing lesions, while previously treated, non-enhancing lesions were omitted, leading to discontinuity in lesion tracking over time.

In the mixed evolution group, which had the highest inconsistency rate, omissions and ambiguities arose from multiple concurrent factors: sequelae lesions were not mentioned, and enlarging lesions exhibited the same issues as in the uniform progression group, being described without precise anatomical localization, without clarification of treatment status, and without temporal correlation.

Narrative reporting concordance was approximately 60% in patients with uniform regression, uniform progression, or new metastatic waves, but dropped sharply to 18.2% in patients with mixed lesion evolution, highlighting that reporting concordance is lowest in the most heterogeneous disease scenarios. Although this subgroup was relatively small, the marked decrease highlights a critical vulnerability of narrative reporting when lesion behavior is heterogeneous.

### 3.3. Reporting Concordance Across Metastatic Waves

We analyzed radiology report concordance in the longitudinal documentation of brain metastases in relation to the number of metastatic waves. A lesion wave was defined as the sequential appearance of new brain metastases during follow-up (Table 2).

Reporting concordance decreased progressively with increasing number of metastatic waves: from 65.2% in patients with a single wave to 46.7% with two waves, 18.2% with three waves, and no concordance in the single case with more than 3 waves. This extreme case illustrates the inherent limitation of narrative reporting: when dozens of lesions appear and evolve across multiple treatment waves, it becomes increasingly difficult to determine which lesions were previously treated, which represent new disease, and which correspond to recurrences. Narrative reporting reaches a saturation point beyond which lesion-level continuity can no longer be reliably preserved.

When considered together with the dimensional evolution patterns (Table 1), these findings highlight that both temporal heterogeneity (new metastatic waves) and morphological heterogeneity (mixed evolution) reduce reporting concordance.

## 4. Discussion

Our study shows that more than half of the tracked lesions either regressed, evolved into post-treatment sequelae, or disappeared completely, reflecting a favorable local response to therapy [8]. However, in patients who developed new lesions, radiology reports often shifted focus almost entirely to the newly enhancing metastases, with previously treated and currently inactive lesions omitted. While this approach may reflect clinical urgency, it risks losing the broader context of disease evolution. Recognizing that previously treated lesions have completely disappeared or stabilized as sequelae is important, as it provides feedback on treatment efficacy.

As the number of metastatic waves and therapeutic episodes increases, maintaining consistency in lesion tracking becomes progressively more challenging. The simultaneous presence of regressing, stable, and progressing lesions within the same examination further amplifies the risk of misclassification or underreporting. Quantitatively, the likelihood of incomplete or ambiguous reports was more than twice as high in patients with mixed dimensional evolution compared with those showing uniform lesion behavior (81.8% vs. mean 39.4%). Similarly, patients with three or more metastatic waves had more than double the inconsistency rate of those with a single wave (81.8% vs. 34.8%). These findings indicate that when lesions have different growth patterns or appear in multiple waves over time, the risk of losing lesion identity increases substantially. This challenge is particularly relevant for lesions treated with stereotactic radiotherapy, which often regress to sub-centimetric residues or evolve into post-treatment sequelae. Such lesions may later develop post-radiotherapy changes, including transient enhancement or radiation necrosis, making longitudinal identity essential for accurate interpretation [9].

The loss of lesion continuity across time represents a critical limitation in current clinical practice. Once treated or regressed lesions are no longer mentioned, and new lesions are described without clear reference to earlier findings, narrative radiology reports provide only a fragmented snapshot of disease evolution. This makes it difficult to reconstruct the cumulative disease trajectory or fully assess therapeutic impact [10].

Notably, lesions treated with stereotactic radiotherapy were not monitored more consistently in narrative reports; once they regressed to post-treatment sequelae, they were often omitted, and in several cases later re-enhanced, making the loss of longitudinal identity clinically relevant. In practice, reconstructing a complete longitudinal history may require reviewing more than ten prior examinations for a single patient, a task that becomes impractical in routine settings [4].

As shown in Table 1 and Table 2, reporting concordance declines with both quantitative complexity (increasing numbers of metastatic waves) and qualitative complexity (heterogeneous lesion evolution patterns), suggesting that both contribute independently to longitudinal reporting vulnerability. In some patients, reporting discordance was driven by missing or incomplete reports rather than interpretative ambiguity; however, these mechanisms frequently overlapped. Once longitudinal context was lost, subsequent reports often became more generic or focused predominantly on clearly identifiable active lesions. Missing documentation itself therefore represents a clinically relevant form of longitudinal information loss. Structured longitudinal tracking systems, particularly if AI-assisted, could complement routine reporting by maintaining coherent lesion histories across time points, reducing ambiguity, and supporting more reliable, patient-centered decision-making without replacing radiologist judgement [11].

Although the direct clinical impact was not evaluated in this study, discontinuous reporting may affect patient care by introducing uncertainty regarding lesion history and treatment status, making it difficult to differentiate late treatment-related effects from new lesions, increasing reliance on retrospective image review, and challenging consistent longitudinal assessment in complex disease courses.

Our observations are consistent with the findings of Machura et al., who demonstrated that ensemble deep-learning approaches can preserve lesion identity across multiple examinations and accurately reconstruct disease evolution in longitudinal MRI studies, while also highlighting the limitations of current clinical reporting, which is often time-consuming, operator-dependent, and insufficiently reproducible [12].

Based on our findings, specific recommendations for improving longitudinal follow-up can be formulated. At a minimum, follow-up reports should explicitly reference previously identified lesions, indicate whether they have been treated, and document their subsequent status (regression, stability, progression, or sequelae), even when no longer enhancing. Additionally, the chronological appearance of new metastatic waves should be clearly distinguished from changes in pre-existing lesions. These elements represent well-defined, repetitive components of reporting that could be supported by structured templates or AI-assisted longitudinal tracking systems [13]. This represents a step toward more accurate, personalized and coherent neuro-oncologic care. More broadly, longitudinal imaging has been highlighted as a key component of outcome modeling in oncology, with the potential to better capture dynamic disease profiles and inform treatment decisions when supported by standardized methods and robust validation [14]. Even with frequent imaging and effective therapies, the absence of structured lesion tracking in routine reports can result in fragmented follow-up and loss of clinically meaningful information.

To our knowledge, no previous studies have specifically addressed the issue of lesion continuity loss in routine radiology reporting of brain metastases. Unlike previous studies that focused on lesion count or volumetric changes [15], our study systematically tracks individual lesions across multiple metastatic waves and evaluates reporting consistency over time.

This study has several limitations. First, the retrospective and single-institution design may limit generalizability. Second, lesion tracking and longitudinal adjudication were performed manually by a single neuroradiologist. While this ensured internal consistency and a stable reference standard, it introduces potential observer bias and limits assessment of inter-reader variability. Third, external MRI studies were acquired with heterogeneous protocols and variable report availability, although all met minimum resolution criteria for inclusion. Fourth, the precision of subgroup analyses was limited by small sample sizes. Finally, we did not evaluate the clinical impact of reporting inconsistencies on management decisions, which should be explored in future studies.

## 5. Conclusions

In this study, we found that routine narrative MRI follow-up reports often provide a fragmented account of lesion evolution in patients with brain metastases. As disease trajectories become more complex and new metastatic waves emerge, the longitudinal continuity of lesions is not consistently documented, limiting the ability to reconstruct a coherent history of therapeutic response.

This study highlights critical gaps in the real-world monitoring of brain metastases and may serve as a foundation for developing longitudinal tracking systems and standardized reporting protocols.

## Figures and Tables

**Table 1:** Reporting concordance of narrative radiology reports stratified by lesion evolution pattern.

Evolution Pattern	Patients (n)	Concordant Reports (n)	Reporting Concordance% (95% CI)
Uniform regression	34	20	58.8% (42.2–73.6)
Uniform progression	16	10	62.5% (38.6–81.5)
New lesion wave	12	7	58.3% (32–80.7)
Mixed evolution	11	2	18.2% (5.1–47.7)

**Table 2:** Reporting concordance of narrative radiology reports stratified by number of metastatic waves.

Number of Metastatic Waves	Patients (n)	Concordant Reports (n)	Reporting Concordance% (95% CI)
1 wave	46	30	65.2% (50.8–77.3)
2 waves	15	7	46.7% (24.8–69.9)
3 waves	11	2	18.2% (5.1–47.7)
>3 waves	1	0	0%

## Data Availability

The datasets analyzed in this study are not publicly available due to patient confidentiality and institutional data protection policies. Reasonable requests for anonymized data may be considered by the corresponding author and the institution, in compliance with applicable ethical regulations.
